# Supplementary material for: IgG1 Is the Optimal Subtype for Treating Atherosclerosis by Inducing M2 Macrophage Differentiation, and Is Independent of the FcγRIIA Gene Polymorphism
Source: Int J Mol Sci. 2023 Mar 21;24(6):5932. doi: 10.3390/ijms24065932 (PMC10053586; doi:10.3390/ijms24065932)
Supplement: Supplementary file 1 [file ijms-24-05932-s001.zip › ijms-2278248-supplementary.pdf]

Supplementary Figures

Supplementary Figure 1 Each DNA fragment obtained by PCR. A. The mapping of heavy chain plasmid. B. The mapping of enzyme cut site. C. 1.0% agarose gel electrophoresis to identify the PCR amplified clones IgG2, IgG3, IgG4 heavy chain target genes and pcDNA-Cγ vector fragments, M: DL5000 DNA Marker.

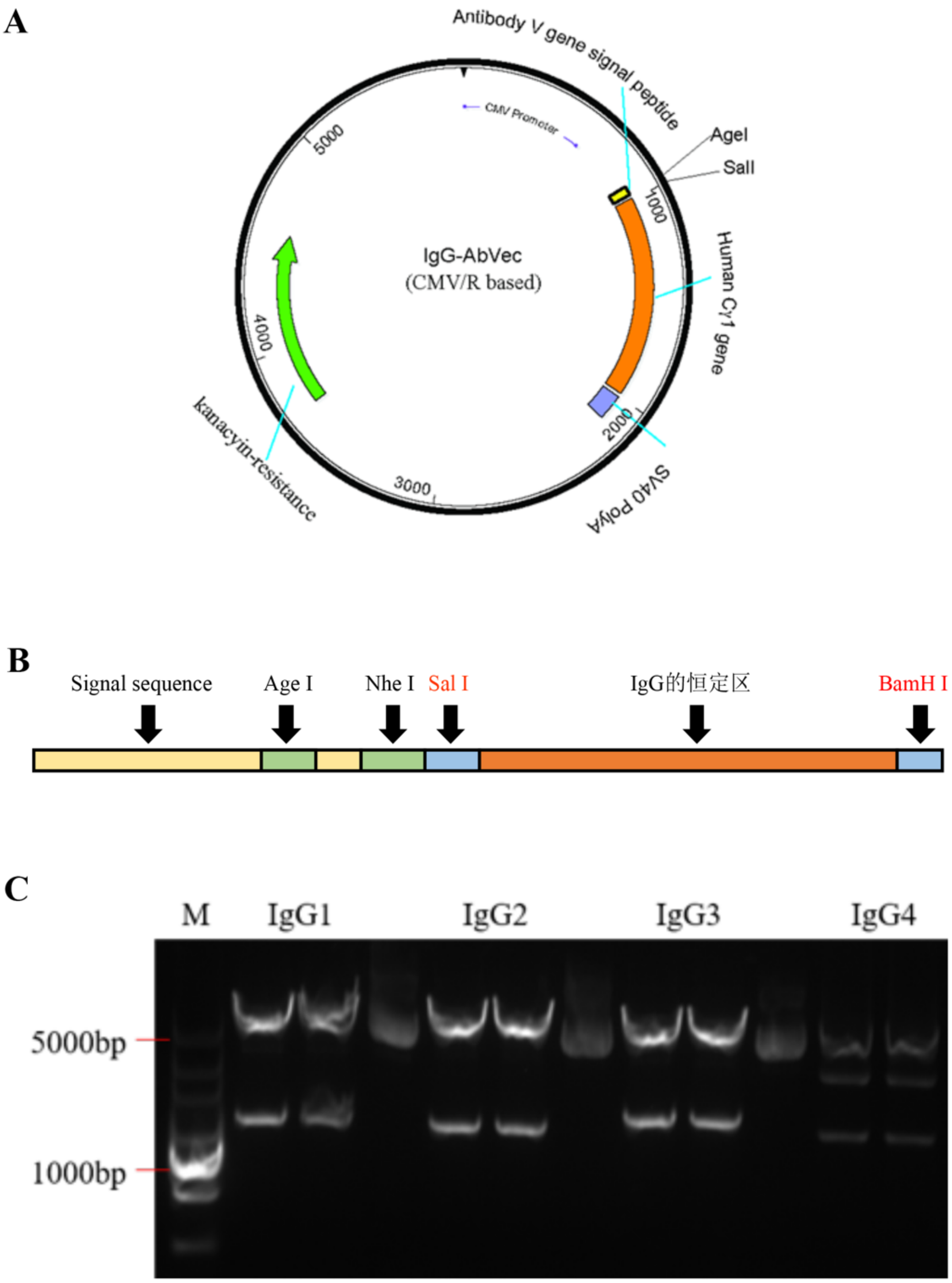

Supplementary Figure 2 The purity and specific affinity of CVI recombinant antibodies of different subtypes and CVI-Fc-engineered antibodies. A. the CVI recombinant antibodies of different subtypes run on a 12% PAGE gel under reducing conditions and were stained by Coomassie Brilliant Blue; B. CVI-Fc-engineered antibodies run on a 12% PAGE gel under reducing conditions and were stained by Coomassie Brilliant Blue; C. Detecting the affinity of CVI recombinant antibodies of different subtypes to COL6A6 via ELISA; D. Detecting the affinity of CVI-Fc-engineered antibodies to COL6A6 via ELISA.

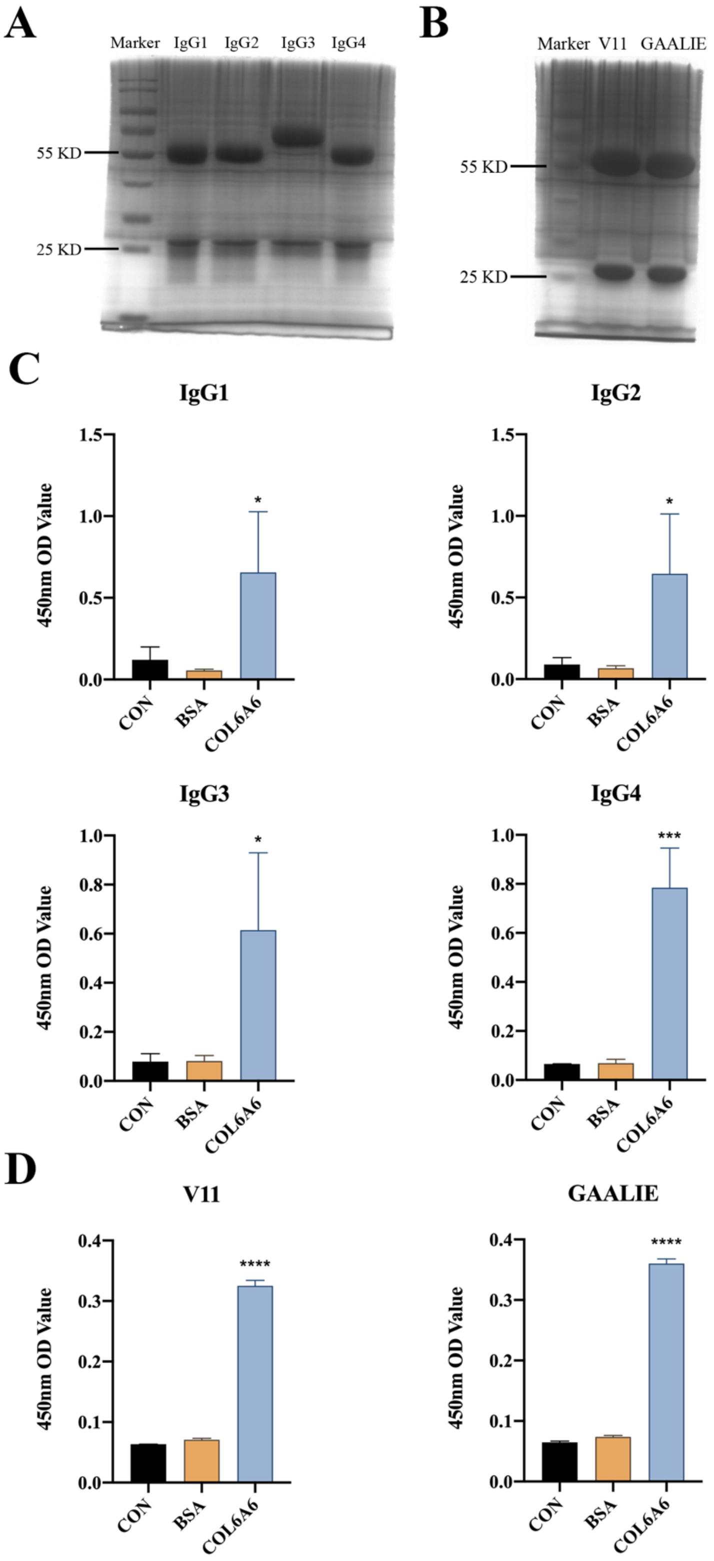

A
Marker IgG1 IgG2 IgG3 IgG4
55 KD
25 KD
B
Marker V11 GAALIE
55 KD
25 KD
C
IgG1
IgG2
IgG3
IgG4
450nm OD Value
CON
BSA
COL6A6
*
***
D
V11
GAALIE
****

Supplementary Figure 3 Snap Gene validates Fc-engineered antibodies.

A. The mutant site of V11. B. The mutant site of GAALIE.

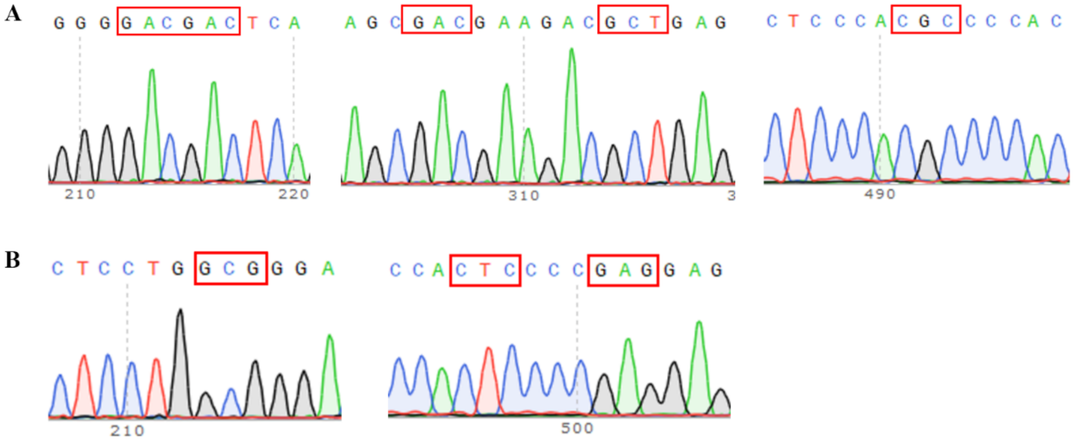

Supplementary Figure 4 The identification of FcγRIIA genotype. A. When the genotype of FcγRIIA is 131 R/R, the DNA is CGT; B. When the genotype of FcγRIIA is 131 H/H, the DNA is CAT; C. When the genotype of FcγRIIA is 131 H/R, the DNA is C(A/G)T.

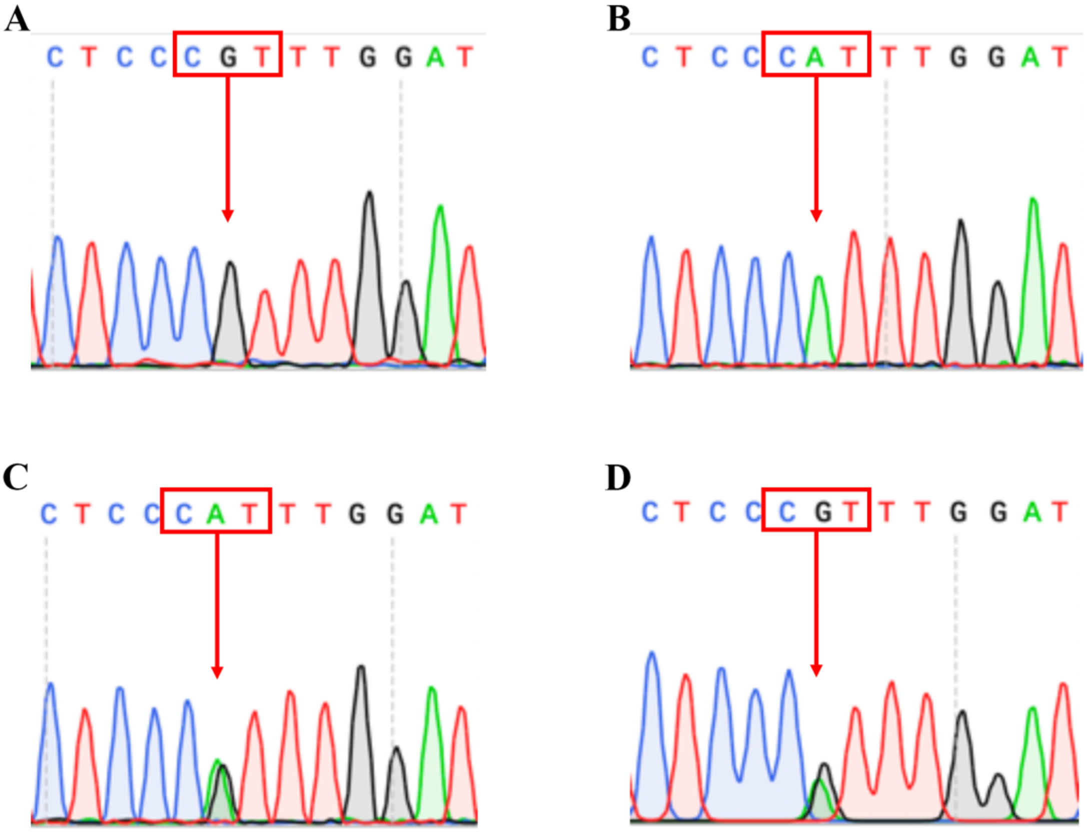

Supplementary Table 1 The statistic of sample size for FcγRIIA genotype.

Name		Genotype			Total	χ^2	*P*
		HH	RR	HR			
Group	Normal	21(52.50)	17(56.67)	29(48.33)	67(51.54)		
	Patients	19(47.50)	13(51.67)	31(43.33)	63(48.46)	0.577	0.749
Total		40	60	30	130		

Supplementary Table 2 The endotoxin's concentration of purified antibodies.

Antibodies	Endotoxin content before endotoxin removal（EU/ml）	Endotoxin content after endotoxin removal（EU/ml）
**IgG1**	4.941	<0.1
**IgG2**	2.918	<0.1
**IgG3**	2.759	<0.1
**IgG4**	3.450	<0.1
**V11**	27.184	<0.1
**GAALIE**	26.304	<0.1
